# Fused oxazepine-naphthoquinones as novel cytotoxic agents with diverse modes of action in yeast

**DOI:** 10.1016/j.heliyon.2024.e41105

**Published:** 2024-12-10

**Authors:** Laura Anaissi-Afonso, Silvia Santana-Sosa, Isabel Lorenzo-Castrillejo, Grant McNaughton-Smith, Félix Machín

**Affiliations:** a Unidad de Investigación, Hospital Universitario Nuestra Señora de Candelaria, Instituto de Investigación Sanitaria de Canarias (IISC), 38010, Santa Cruz de Tenerife, Spain; b Instituto de Tecnologías Biomédicas, Universidad de La Laguna, 38200, San Cristóbal de La Laguna, Spain; c Centro Atlántico del Medicamento S.A. (CEAMED S.A), 38204, La Laguna, Spain; d Facultad de Ciencias de la Salud, Universidad Fernando Pessoa Canarias, 35450, Las Palmas de Gran Canaria, Spain

**Keywords:** Benzoxazepine, naphthoquinone, *Saccharomyces cerevisiae*, Oxidative stress, cell cycle, Nucleolus

## Abstract

The naphthoquinone moiety is commonly found in numerous natural cytotoxic compounds with diverse and pleiotropic modes of action (MOAs). The moiety can exist as a standalone pharmacophore or combined with other pharmacophores to enrich their MOAs. Here, we report that the synthetic fusion of naphthoquinones and oxazepines provides potent cytotoxic compounds with diverse MOAs. Fused oxazepine-naphthoquinones were identified through a cytotoxic screen in *Saccharomyces cerevisiae*. The two most active compounds, CM-568 and CM-728, contained the same 3-pyridyl appendage in the oxazepine ring and were further evaluated along with close chemical derivatives. Both were highly cytotoxic, killing yeast cells in the low micromolar range; however, the role of reactive oxygen species in their MOA was significantly different. Investigations with yeast strains specifically designed to assess cell cycle, chromatin compaction, and nucleolar activity suggest that at lethal concentrations, cells die shortly after drug exposure through programmed death. Conversely, at sublethal concentrations, cell cycle progression is severely impaired. Interestingly, CM-568 labels cells with highly refractive non-fluorescent parallel rods. We conclude that the oxazepine moiety confers novel cytotoxic MOAs to naphthoquinones, which may be potentially useful in pharmacology.

## Highlights

• Fused oxazepine-naphthoquinones are cytotoxic against yeast.
• The oxazepane ring with a 3-pyridyl appendage gives the most potent activity.
• Modifications in the naphthoquinone ring shift the mode of action.
• Oxidative stress and cell inclusions are observed during exposure.
• There is no cell cycle arrest, but nucleolus and chromatin shrink.

## Graphical abstract

**Figure:**
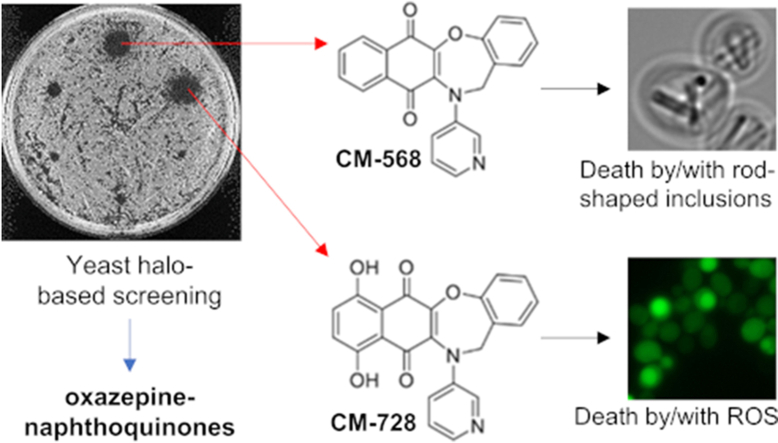

## 1 Introduction

The naphthoquinone group is present in numerous natural products. Naphthoquinones (NQs) generally display cytotoxic properties through various non-exclusive mechanisms [1]. They can act as catalysts in redox cycles powered by NAD(P)H and molecular oxygen, which results in the generation of harmful reactive oxygen species (ROS). On the other hand, naphthoquinones with unmodified carbons in the quinone ring can be powerful electrophiles that react and form new covalent bonds with nucleophilic atoms in biomolecules. Examples of such nucleophiles within cells are reactive thiol groups in enzyme catalytic centers, as well as reactive amine groups in DNA and the microtubule cytoskeleton [2,3]. In addition, they can also act as more specific and less reactive enzyme inhibitors [4]. Despite their pleiotropic modes of action (MOAs), many natural NQs show cytotoxic selectivity against pathogenic bacteria, protozoa, fungi, and cancer cells [[5], [6], [7], [8]]. These selective properties confer biopharmaceutical potential to NQs. Other moieties present in the NQ containing molecule may modulate, and even shift, the MOAs. These additional moieties can also target NQs against specific biomolecules.

The benzoxazepine moiety is also present in active compounds against bacteria, protozoa, fungi, and cancer cells [9]. The antitumor properties of benzoxazepines are mediated by several cellular targets, including microtubules [10,11]. In fact, benzoxazepines define a novel class of anti-tubulin agents distinct from the two major classes that comprise the widely used chemotherapeutic drugs based on vinca alkaloids and taxols, respectively [10].

The yeast *Saccharomyces cerevisiae* is a powerful tool in cell-based assays aimed at identifying promising drug candidate and understanding their MOAs [12,13]. The organism is suited for drug screening, even when the amount of compound is a serious limitation. In addition, chemical genetics through comparison between isogenic mutant strains, as well as growth under distinct physiological conditions, facilitate the delimitation of MOAs [14,15]. In the particular case of NQs, *S. cerevisiae* can be grown anaerobically, which blocks putative redox cycles, and thus the deleterious consequences of both NAD(P)H depletion and ROS generation [6,16]. Moreover, cells further counteract ROS through the oxidative stress response (OSR), which comprises a plethora of buffering and repair mechanisms that are exquisitely regulated by Yap1 [17]. Thus, yeast devoid of Yap1 (Δ*yap1*) are hypersensitive to ROS. Likewise, ROS-driven secondary damage to DNA and microtubules is buffered by other conserved mechanisms, such as the DNA damage response (DDR) and spindle assembly checkpoint (SAC), respectively [18,19]. Direct and indirect damage to these two important targets can be easily established as MOAs by comparing cells proficient and deficient for DDR or SAC. For example, Δ*mad2* strains (which disables SAC in yeast) are sensitive to drugs that target microtubules, while a yeast strain with the double mutant Δ*rad9* Δ*rad52* (ΔΔrad), which abolishes an efficient DDR, is sensitive to DNA damaging agents. Cell organelles can also be specifically targeted by drugs, and several NQs appear to selectively compromise the mitochondrial respiratory chain, rendering yeast cells unable to grow on non-fermentable carbon sources [6]. In yeast, this can be easily investigated by comparing the growth inhibition when employing glucose versus glycerol as the carbon source. Alternatively, NQs can be either inactivated or hyperactivated by respiratory chain oxidoreductases, scenarios that can be easily tested using respiration-deficient (*rho-*) yeast strains. Finally, NQ toxicity can be attenuated by the cell pleiotropic drug resistance (PDR) systems, which is largely impaired in a quadruple mutant strain (ΔΔΔΔpdr) that carries knockout deletions for *PDR1*, *PDR3*, *YRR1*, and *YOR1* [20].

Here, we have applied this powerful combined screening platform to a collection of novel fused oxazepine-naphthoquinones compounds. Two highly cytotoxic compounds, CM-568 and CM-728, were identified from this screening process. Further screening of close chemical analogues revealed that the fused oxazepine-naphthoquinones system was the key to high potency. We further found that despite similar potencies in yeast, the two most active compounds possessed significantly different MOAs. Interestingly, bright field analysis of cells treated with CM-568 produced a cell sign that, to the best of our knowledge, has not been reported before.

## 2 Materials and methods

### 2.1 Compounds and reagents

All tested compounds (n = 145) were supplied by CEAMED S.A. as 10 mM stocks in DMSO in 96-well plates. A duplicate 96-well plate with 1 mM stocks in DMSO was then prepared, and both stocks were used in the screen. Their synthesis is disclosed in European patent application EP21382995 and will be published elsewhere. The purity of the compounds was determined to be >95 % by high pressure liquid chromatography (HPLC). Purity and characterization details of CM-568 and CM-728 are provided in supplemental information (Figs. S1–S4). HPLC was performed using a Jasco PU-2080 intelligent HPLC pump (Jasco MD 2020 Plus multiwavelength detector). ^1^H and ^13^C NMR spectra were recorded at room temperature on a Bruker Avance 500 MHz NMR spectrometer using deuterated chloroform (CDCl_3_) as solvent. High-resolution mass spectrometry (HRMS) was performed on a Micromass AutoSpec magnetic tri-sector (EBE geometry) mass spectrometer.

Other reagents used were dimethyl sulfoxide (DMSO; Sigma-Aldrich, #5895690100), propidium iodide (PI; Fluka, #81845), and 2′-7′-dichlorofluorescin diacetate (DCFH-DA; Sigma-Aldrich, #D6883).

### 2.2 Yeast strains

The wild-type reference yeast strain was the *MAT*a haploid BY4741. All single mutants were commercial isogenic derivatives of BY4741 obtained from the Euroscarf collection (http://www.euroscarf.de). The Δ*rad9* Δ*rad52* (ΔΔrad) double mutant and the mitochondrial respiratory chain-deficient BY4741 derivative (*rho0* genotype) have been described before [6]. The quadruple mutant strain deficient for the pleiotropic drug resistance (ΔΔΔΔpdr; Δ*pdr1* Δ*pdr3* Δ*yrr1* Δ*yor1*) was provided by the NBRP, Japan [20]. All strains were grown overnight at 25 °C in the rich medium YPD (1 % w/v yeast extract, 2 % w/v peptone, 2 % glucose) until log-phase (0.3–0.8 OD_620_). In the case of plates, 2 % w/v agar was added to the YPD. YPgly plates contain 2 % w/v glycerol instead of glucose.

### 2.3 Halo and dose-response inhibition assays

Inhibition halo assays were performed as previously described [6], with a cell density on the plate surface of ∼50 cells/mm^2^. The amount spotted for each compound was either 1 or 10 nmol. Inhibition halo assays were also used to check the effects of hypoxia and glycerol, as the assay overcomes the caveats of lower growth rates in these cases. In general, photos of halos were taken every 24 h for 3 d, except for hypoxia and glycerol, which were taken only after 72 h. Hypoxia was achieved by placing the YPD plate into a sealed bag of the Anaerocult™ A mini kit (Merck, #1.01611.0001).

To quantify inhibition in the halo screening, a circle of 10 mm of diameter was drawn around the spot using Fiji/ImageJ (https://imagej.net/software/fiji/), and the overall intensity of the area (∼78.5 mm^2^) was measured and normalized to an equivalent non-halo area near the centre of the plate (set as 1) and an equivalent black area outside the plate (set as 0). Quantification of inhibition in the other halo assays was based on the diameter of the halo.

The dose-response inhibition assay was performed in 96-well plates as previously described [6]. Drug concentrations ranged 1–128 μM in 1:2 serial dilutions plus a DMSO control. The final concentration of DMSO was 1 % v/v. The inoculum was adjusted to an optical density at 620 nm (OD_620_) of 0.001. Growth was measured at OD_620_ after 48h at 25 °C.

### 2.4 Biocidal assay

A variation of a dose-response clonogenic spot assay was used to determine whether the compounds killed yeast cells. Strains were grown overnight to log-phase, diluted to an OD_620_ of 0.1, and 0.1 mL incubated for 3h at 30 °C with different doses of the tested compounds (1.5–100 μM range in seven 1:2 serial dilutions; an eighth subculture was treated with DMSO 1 % v/v). Incubations were carried out in tilted 1.5 mL tubes at 180 rpm. After drug treatments, subcultures were further diluted to 0.01 OD_620_ and six 1:3 serial dilutions were prepared in a 96-well plate, resulting in an 8x6 matrix. This matrix of samples was spotted onto YPD plates using a 48-pin steel replicator (Sigma-Aldrich, #R2383). The plates were photographed after 3 d at 30 °C.

### 2.5 Fluorescence microscopy

The strain FM2707 (*MATa Δbar1 cdc15-2 ADH1p-OsTIR1 TetR-YFP cXIIr-Tel(*1061 Kb*):tetO(x224) HTA2-mCherry NET1-eCFP*) was used for the short-term profile at the cell biology level upon drug exposure. The origin and construction of the strain has been reported before [21]. The strain was grown to log-phase in YPD (25 °C), and a G1-to-telophase synchronized cell cycle was evaluated under different concentrations of the tested drugs as reported before [22]. The G1 arrest was achieved by incubating with 50 ng/mL α-factor for 3h at 25 °C, and the G1-to-telophase was monitored 3h after the removal of α-factor. Cells were incubated at 34 °C after α-factor removal to arrest cells in the ensuing telophase by means of the *cdc15-2* thermosensitive allele.

Individual cells were visualized in a Leica DMI6000B microscope with an ultrasensitive DFC350 digital camera under the 63X/1.30 immersion objective, as reported before [21]. A series of 8 z-slices (0.5 μm depth) were taken around the focal plane and flattened into a two-dimensional maximum projection using the Fiji/ImageJ software.

### 2.6 Determination of ROS

The strain BY4741 was used for ROS determination as reported before with minor modifications [22,23]. A log phase culture grown on YPD was diluted to an OD_620_ of 0.1, and split in three 1 mL subcultures that were incubated with DMSO 1 % v/v, 100 μM CM-568 and 100 μM CM-728, respectively. At different time points (0.5h, 1h and 2h), an aliquot of 300 μL was taken and incubated with 10 μg/mL DCFH-DA and 3 μg/mL PI for 15’ in the dark. The cells were photographed and processed as described above. For ROS quantification, the cell area of all PI-negative cells in a field was selected and the mean intensity for each cell recorded with Fiji/ImageJ.

### 2.7 Data representation and statistics

Population-based data (cell cycle profiles, growth inhibition in halo assays, and dose-response curves) were presented in either XY or scatter dot plots. Error bars in dot plots represent the standard error of the mean (sem), whereas the horizontal line represent the mean. Data based on single cell analysis (ROS, nucleolar and chromatin area) were presented in box plots. In these plots, the horizontal bar represents the median, the box delineates the 25th and 75th percentiles, the whiskers extend to Tukey's interquartile range, and the dots represent the Tukey's outliers. More than 150 cells per sample were quantified. At least two independent biological replicates were performed for each assay.

One-way ANOVA followed by Tukey's post hoc was used for comparisons of ROS and nucleolar/chromatin areas. For inhibition halos, the Dunnett's post hoc was used instead to compare either mutants or physiological conditions with the corresponding control.

GraphPad Prism 10 was used for both graphical display and statistical analysis.

## 3 Results

### 3.1 Identification of fused oxazepine-naphthoquinones as cytotoxic drugs in a halo-based yeast screen

**Fig. 1:**
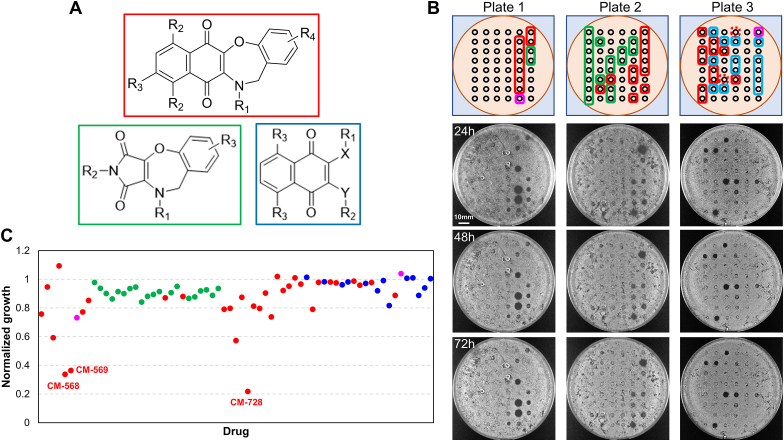
**Yeast toxicity screening of oxazepine-naphthoquinones and related compounds.** (**A**) Schematic of the main families of compounds tested. In red, oxazepine-naphthoquinones; in green, oxazepine-pyrrolediones; in blue, oxazepine-naphthoquinones-like molecules where the oxazepine ring is broken. (**B**) Toxicity screening by halo inhibition assay (1 nmol spots). At the top, scheme of the compound position on plates. Red solid lines, oxazepine-naphthoquinones; red dashed lines, diazepine-naphthoquinones; pink lines, oxazepine-quinoline‐5,8‐diones. (**C**) Quantification of halo inhibition. The three most potent compounds are named.

We previously used an inhibition halo assay to characterize MOAs of naphthoquinones [6]. This assay has the advantage of forming a concentric concentration gradient of the drug, which simplifies dose-response exploration during screening and chemical genetics studies. In the screen, the wild-type reference *S. cerevisiae* haploid strain BY4741 was grown as a lawn on a rich medium (YPD) plate, and 47 potential drugs were spotted at defined positions, leaving a 48th spot for the vehicle control (DMSO in most cases) (Fig. S5). While screening plates of compounds from different sources, we identified three oxazepine-naphthoquinone compounds, distributed over three contiguous plates, that gave clear permanent inhibition halos in BY4741 (Fig. 1A and B), with normalized growth around the spot of less than 0.5 relative to DMSO (Fig. 1C; Tables S1–S4). These three oxazepine-naphthoquinones (CM-568, CM-569, and CM-728) belonged to a larger group of diazepine- and oxazepine-naphthoquinones (N = 32; red labels), which in turn were closely related to other compounds where the naphthoquinone moiety has been replaced by either a pyrroledione ring (N = 20; green labels) or a quinoline‐5,8‐dione (N = 2; pink labels). In addition, there were other compounds that can be envisioned as naphthoquinones where the oxazepane ring has been opened (N = 13; blue labels). The other compounds on the three plates were also from CEAMED but were chemically unrelated.

### 3.2 Structure activity relationships from wild type (BY4741) yeast assays

Based on the levels of activities demonstrated by these compounds, rapid initial structure-activity relationships (SAR) could be established. Firstly, compounds bearing the naphthoquinone ring system connected to the oxazepane (red) were in general more active compared to those with the pyrroledione ring system (green). Furthermore, naphthoquinones compounds without the oxazepine fused system (blue) were generally not active (Fig. 1A and C). CM-568, CM569 and CM-728 share the presence of a pyridyl moiety attached to the oxazepane ring; however, this alone is not enough to confer yeast cytotoxicity as three other oxazepine-naphthoquinones contain similar attachments and were poorly active in the halo assay (CM-784, CM-785 and CM903; Table S1).

**Fig. 2:**
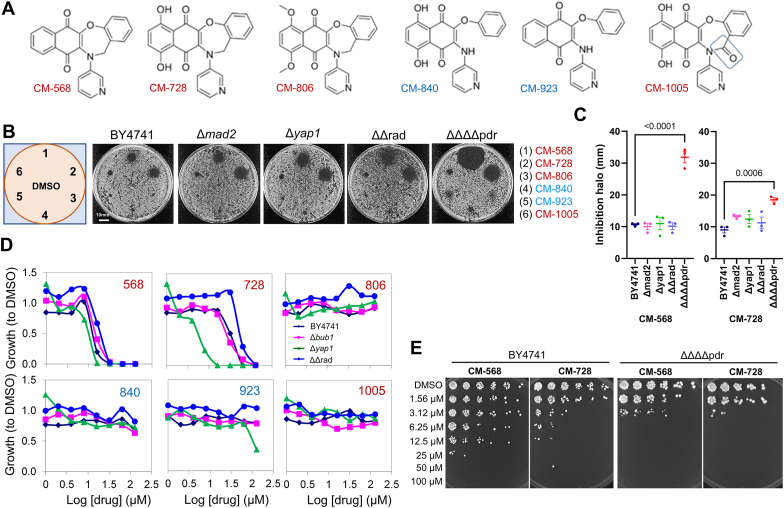
**Common modes of action, drug resistance and biocidal profiles of selected oxazepine-naphthoquinones.** (**A**) Schematic of six compounds selected for further studies. The four compounds on the right are CM568 and CM-728 derivatives not included in the original screening. In red, oxazepine-naphthoquinones; in blue, oxazepine-naphthoquinones-like molecules with a broken oxazepine ring; the blue rectangle indicates the added ketone group in CM-1005. (**B**) Growth inhibition halos (10 nmol spots) for the reference strain BY4741, the SAC mutant *mad2*Δ, the OSR mutant *yap1*Δ, the DDR double mutant *rad9*Δ *rad52*Δ (ΔΔrad), and a PDR quadruple mutant (ΔΔΔΔpdr). (**C**) Quantification of halo diameters for CM-568 and CM-728 (mean ± sem, n = 3); p values are shown for the pairwise comparisons (one-way ANOVA followed by Dunnett's post hoc test). (**D**) Dose-response growth inhibition curves (48h) for BY4741, SAC mutant *bub1*Δ, OSR mutant *yap1*Δ, and DDR double mutant *rad9*Δ *rad52*Δ (ΔΔrad). Growth was normalized to the vehicle (DMSO 1 % v/v). (**E**) Clonogenic assay for cell survival after 3h exposure to CM-568 and CM-728.

To further investigate the SAR, a series of specific analogues of the two most potent compounds, CM-568 and CM-728, were added (Fig. 2A). These compounds were not included in the initial screening, and were thus tested in the halo assay together with CM-568 and CM-728 (Fig. 2B and C). CM-568 and CM-728 contain the same naphthoquinone-oxazepine fused ring system and contain the same 3-pyridyl appendage. They differ in that CM-728 has an additional two aromatic hydroxyl groups positioned adjacent to the two carbonyl groups of the naphthoquinone ring. In the wild type yeast (BY4741) both compounds form halos of significant and comparable sizes. Compound CM-806 also contains the naphthoquinone-oxazepine fused ring system as CM-728, but instead of hydroxyl groups it has two methoxy groups. This addition of the extra methyl groups was very detrimental to the activity of the compound as no noticeable inhibition of growth was observed. Similarly, the addition of another carbonyl group, this time adjacent to the nitrogen in the oxazepine system (CM-1005) also caused a great reduction in activity. The chemical changes in both CM-806 and CM-1005 will dramatically affect the electron-distribution in the quinone system, and this may be the cause of the lack of activity seen. Furthermore, ring open versions of CM-568 and CM-728, CM-923 and CM-840 respectively, were also shown to have little inhibitory activities, highlighting the favourability of the more planar fused ring system. These SAR results in the halo assays with the wild type yeast were repeated in dose-response assays in liquid cultures, in which only compounds CM-568 and CM-728 were active (Fig. 2D). These concentration-response curves also indicated that CM-568 was slightly more potent than CM-728 (GI_50_ of ∼15 and 30 μM, respectively).

### 3.3 CM-568 and CM-728 MOA studies using genetically modified yeast strains

Together with BY4741, several isogenic mutants were tested to identify common MOAs of NQs and oxazepines. Thus, we included mutant strains sensitive to drugs that target microtubules (Δ*mad2* or Δ*bub1*), act as DNA damaging agents (ΔΔrad), and/or cause oxidative stress (Δ*yap1*).

Although the halo assays did not reveal a significant increase in sensitivity for any of the MOAs explored (Fig. 2B–D), they did reveal that when pleiotropic drug resistance was lowered (ΔΔΔΔpdr) CM-568 was twice as potent as CM-728. Interestingly, concentration-response growth curves in liquid culture showed a hypersensitivity of the Δ*yap1* strain to CM-728 but not CM-568 (Fig. 2C).

### 3.4 CM-568 and CM-728 are biocidal against yeast

To test whether the observed growth inhibition was due to a cytostatic or biocidal effect, asynchronous log-phase cultures of BY4741 and ΔΔΔΔpdr strains were exposed briefly (3h) to increasing concentrations of the drugs. After the short exposure, cell survival was assessed through a spot clonogenic assay (Fig. 2E). The results for BY4741 indicate a biocidal effect above 10 μM for CM-568 and 3 μM for CM-728. As expected, this biocidal effect was observed at even lower concentrations in the ΔΔΔΔpdr strain (∼1.5 μM). Interestingly, CM-728 was more biocidal than CM-568, despite the latter being more efficient at stalling cell growth. This suggests that CM-568 may have both cytostatic and biocidal effects.

### 3.5 CM-728, but not CM-568, is an oxidative stressor that affects mitochondrial function

**Fig. 3:**
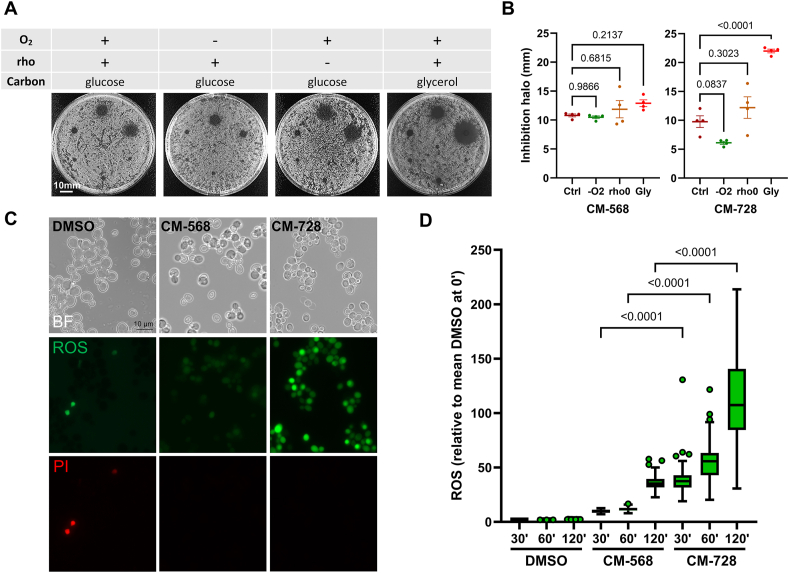
**Role of redox cycling and ROS in oxazepine-naphthoquinones toxicity.** (**A**) Growth inhibition halos (10 nmol spots) for BY4741 grown under normoxia and anoxia (after 24h and 72h, respectively). Also shown are normoxic halos for a *rho0* strain derivative and BY4741 grown on YPGly (non-fermentable glycerol, 72h). The same six oxazepine-naphthoquinones shown in Fig. 2A were tested; the order of spots is as in Fig. 2B. (**B**) Quantification of halo diameters for CM-568 and CM-728 (mean ± sem, n = 4) (p values are shown for the pairwise comparisons; one-way ANOVA followed by Dunnett's post hoc test). (**C**) ROS production after 2 h with CM-568 and CM-728 (100 μM). DMSO 1 % v/v was used as control. (**D**) Quantification of ROS at different incubation times with the drugs (p values are indicated for the significant pairwise comparisons; one-way ANOVA followed by Tukey's post hoc test).

As the NQ moiety is present in both CM-568 and CM-728 and can modulate levels of ROS, we decided to further investigate the contribution of ROS to the MOA of these compounds. ROS requires O_2_ as a substrate and is often, but not always, generated by the mitochondrial respiratory chain. Alternatively, NQ-mediated ROS commonly targets the respiratory chain, rendering it inactive [6]. These different scenarios were tested by monitoring growth inhibition in: (i) the presence or absence of O_2_; (ii) in a respiratory deficient (rho-) phenotype; and (iii) a fermentable (glucose) versus a non-fermentable (glycerol) carbon source (Fig. 3A and B). In all systems, the behaviour of CM-568 was similar; however, CM-728 was less active in hypoxia (-O_2_) and more active in glycerol. This profile is consistent with ROS-mediated toxicity, which in turn leads to mitochondrial dysfunction [6]. Interestingly, the source of ROS may be outside the respiratory chain as CM-728 had the same levels of activity in the rho+ and rho- strains*.*

In addition to these chemical genetics and physiological approaches, we measured ROS production after short exposure to each compound. ROS was monitored under the microscope using the fluorescent agent dichloro-dihydrofluorescein diacetate (DCFH-DA) (Fig. 3C and D). The presence of ROS is indicated by a positive signal in the green channel after the low-fluorescent DCFH-DA is converted and accumulated within the cell to the high-fluorescent dichloro-fluorescein (Fig. 3C). At the same time propidium iodide (PI) was added to determine whether the integrity of the plasma membrane was being maintained (a red fluorescence indicating loss of integrity). Under these experimental conditions CM-728 continually increased ROS production over the 2h time period, reaching a maximum of over a 100-fold increase compared to the DMSO control (Fig. 3D). CM-568 also produced ROS, but with a more modest fold increase (∼40-fold after 2h).

These results, together with the Δ*yap1* hypersensitivity to CM-728 (Fig. 2D), strongly suggest that ROS production plays a major role in CM-728 toxicity, and a more minor role in the case of CM-568. Alternatively, futile redox cycling, which boosts ROS production but also depletes cells of reducing agents (NADH/NADPH), may underlie CM-728 toxicity as well.

### 3.6 CM-568 gives rise to intracellular refractive rod-shaped inclusions

**Fig. 4:**
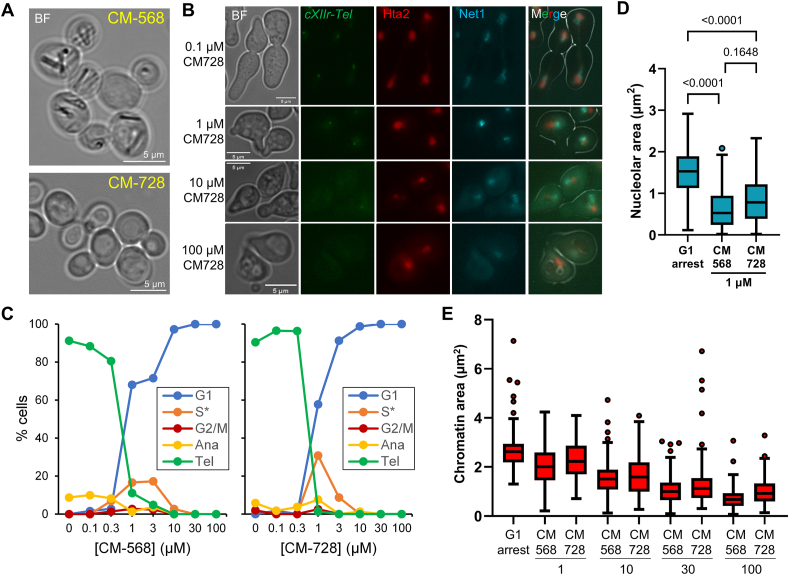
**Short-term effects of CM-568 and CM-728 at the cellular level.** (**A**) Cell appearance after 1h incubation with 100 μM of each drug. Treatment was carried out on BY4741. (**B**) Prototypical FM2707 cells after a G1-to-telophase cell cycle in the presence of increasing concentrations of CM-728. (**C**) Quantification of the G1-to-telophase dose-response profiles. (**D**) Nucleolar area in the 1 μM treatment. Only unbudded G1-like cells were counted and compared to G1 arrest (p values are shown for the pairwise comparisons; one-way ANOVA followed by Tukey's post hoc test). (**E**) Chromatin compaction with increasing drug concentrations. Only unbudded G1-like cells were counted and compared to G1 arrest (see Fig. S7 for statistics).

Short treatments with lethal concentrations of CM-568 and CM-728 blackened a percentage of cells under bright field (BF) microscopy (Fig. 3C). This pattern is suggestive of cell death, although these cells were not labelled with PI. Interestingly, a closer look uncovered a clear difference between CM-568 and CM-728 (Fig. 4A). CM-568 resulted in the striking formation of refractive dark inclusions within the cell, whereas darkened cells in CM-728 were characterized by the formation of large vacuoles and the absence of these inclusions. These morphological patterns were a major difference between these otherwise closely related compounds.

### 3.7 Cell cycle, nucleolar and nuclear profiles under CM-568 and CM-728

In addition to being an excellent model for cell-based screening and chemical genetics, yeast is a powerful tool for studying cell biology in the presence of drugs. Cell biology can determine how a drug affects the cell cycle (i.e., cell cycle arrest indicating checkpoint activation), chromosome segregation, nucleolar activity, and reveal morphological signs of programmed cell death. All of these can be assessed at once in a strain specifically engineered for such purposes. This strain, whose cell cycle can be synchronized at different stages, contains selective fluorescent markers for the bulk of chromatin (Hta2-mCherry), the nucleolus (Net1-CFP), and the last genomic region to segregate in anaphase (chromosome XII right arm telomere; cXIIr-Tel; labelled with the *tetO*/TetR-YFP system) [21].

A log-phase culture of this strain was synchronized in G1, and then released into the cell cycle. The strain also carries the thermosensitive allele *cdc15-2*, which allows monitoring of a single G1-to-telophase cell cycle [22]. G1 cells are easy to identify because they appear as single, unbudded cells with a characteristic pear-like appearance in response to the mating type pheromone α-factor, which induces G1 arrest (Fig. S6A). Cells that have reached telophase after G1 release appear as large budded cells (dumbbell morphology) that have completed segregation of the genetic material (i.e., segregated chromatin, nucleoli and cXIIr-Tel) (Fig. S6A). Between G1 and telophase, the different cell cycle stages are characterized by distinct patterns: S-phase, mononucleated cells with small-medium buds (less than half the size of the mother cell); G2/M, mononucleated cells with medium-large buds (more than half the size of the mother; a large bud the size of the mother indicates G2/M arrest); anaphase, medium-large buds with an elongated nucleus and unresolved cXIIr-Tel.

Short-term exposure (1–2 h) to 100 μM of either CM compound did not show a clear pattern of cell cycle arrest (Fig. 3, Fig. 4A). The G1-to-telophase dose-response experiment showed that cells did not enter the cell cycle at a concentration higher than 10 μM (Fig. 4B and C). At lower concentrations, no G2/M peak was found, ruling out a G2/M arrest [22]. Instead, at around 1 μM, cells were partitioned into those that could not bud and those with a small bud (S∗ category). These small buds do not indicate proper cell cycle arrest in the S phase, which phenocopies that of G2/M block, but rather the inability of the bud to grow further. At lower concentrations (<0.3 μM), cells completed the G1-to-telophase cell cycle (Fig. 4B and C). Interestingly, the intensity signals of both YFP and CFP, but not that of mCherry, were largely diminished at concentrations above 10 μM (Fig. 4B and S6B). The loss of fluorescence occurred at lower concentrations for CM-728 (<10 μM) than for CM-568 (>10 μM).

In addition to aiding in the determination of the cell cycle, the cell area covered by the bulk of the chromatin in general and the nucleolus in particular can report the metabolic and vital state of the cell. A clear shrinkage of the nucleolar area was observed for both CM-568 and CM-728 at 1 μM (Fig. 4D), suggesting a stalling of ribosomal RNA transcription, a sign of metabolic stress [24]. Similarly, a steady shrinkage of chromatin area was observed for both CM compounds (Fig. 4E). This sign is also consistent with metabolic stress and may also indicate programmed cell death at higher concentrations [25].

## 4 Discussion

In this study, we have identified two oxazepine-naphthoquinones, CM-568 and CM-728, that are cytotoxic against yeast (Fig. 1). Using dose-response growth curves (Fig. 2D), halo inhibition (Fig. 1, Fig. 2B,C), and clonogenic assays (Fig. 2E), we conclude that both compounds are biocidal in the low micromolar range, which is impressive in yeast. The cell biology profile of short-term treatments also suggests a common cytotoxic pattern for both compounds: inability of cells to grow the newly formed bud and shrinkage of the nucleolar area at low concentrations (Fig. 4C and D), while chromatin shrinks (Fig. 4E), cells become darker (Fig. 3C), and GFP-based fluorescence is lost at higher concentrations (Fig. 4B).

Despite the similarities we found between CM-568 and CM-728, we also found some striking differences that point towards different MOAs. For example, CM-728 gave a profile consistent with ROS and/or redox cycling being major players in toxicity (Fig. 3). CM-568 did not fulfil that profile but produced an original cell sign consisting of refractive non-fluorescent rod-shaped inclusions (Fig. 3, Fig. 4A).

**Fig. 5:**
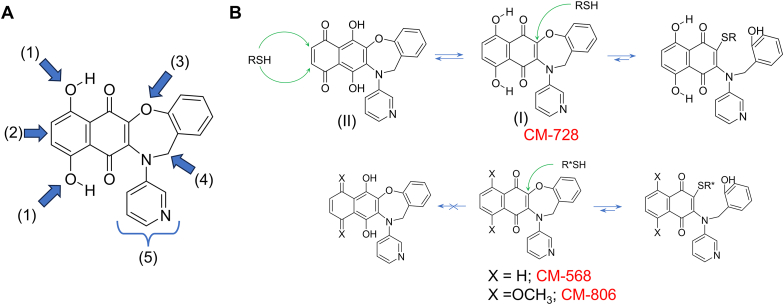
**Structure-activity-relationship (SAR) conclusions and putative mechanism of action on thiol groups.** (**A**) Overview of SAR. CM-728 is depicted to show critical moieties and residues, which are pointed by blue thick arrows and curly brackets: (1) -OH (CM-728) versus -H (CM-568) changes the MOA while keeping overall toxicity; (2) naphthoquinone is more active than pyrroledione system; (3) NH or NCH_3_ are not active; (4) the ring system is favoured compared to non-cyclized systems (CM-840), and -CH2- favoured over CO (CM-1005); (5) Aryl groups with electron withdrawing groups in positions 3 and 4 are favoured (e.g. CN, NO2, or pyridyl), and -H (i.e NH) is not active. (**B**) Possible binding/reaction centers with thiol groups (RSH) in cysteines. (I) and (II) denotes the expected equilibrium between the quinone and the 1,4-phenolic hydroxyl derivative in CM-728.

Consistent with the profile we observed for CM-728, a recent article used chemoproteomics to identify CM-728 as a specific inhibitor of human peroxiredoxin-1 [26]. This enzyme plays an important role in ROS detoxification and thiol group turnover [27]. This compound and its human target have shown promising antitumor potential [26,28]. Many of the mechanisms for maintaining the balance of ROS depend on redox systems involving proteins containing reactive cysteine groups, such as the thioredoxin–peroxiredoxin (TRX-PRDX) systems. A reduction in the levels of these proteins, by their reaction with electrophiles, can lead to a reduction in the cell capability to remove ROS. This was the mechanism proposed to explain why CM-728 rapidly and significantly raises internal ROS in human cell lines [26], and it may also be the mechanism underlying the boost of ROS in yeast. Indeed, the SAR generated from the various chemical modifications we have undertaken suggests that CM-728 possesses several features that would make it a good scavenger of thiol (cysteine) groups (RSH) (Fig. 5). Firstly, it contains a naphthoquinone system which can act as an electrophile for Michael-type addition reactions. Furthermore, the groups attached across the double bond generate an electronic gradient across the double bond (the essential electron-withdrawing group on the N atom and the electron donating capability of the phenoxy oxygen), increasing its electrophilic nature. The oxazepine ring system is also a favourable entity. The better leaving group capability of the phenoxy oxygen compared to inactive diazepines (Table S2) is also consistent with a possible RSH addition-elimination mechanism. Moreover, the fact that the oxazepine system was more active than the open ring versions also agrees with a possible RSH reactive mechanism as the flatter oxazepine ring will allow for a less hindered attack on the double bond carbon compared to the open systems. Finally, the 1,4-phenolic hydroxyl groups adjacent to the carbonyl groups in CM-728 could also be important for its distinct MOA, as it has been suggested for the difference between the dihydroxylated naphthoquinone shikonin and its non-hydroxylated analogues [29]. On the one hand, the hydroxyl groups form internal hydrogen bonds with their neighbouring carbonyl group, increasing the electrophilic nature of the OCC=CCO Michael acceptor system, and on the other hand, the 1,4-phenolic hydroxyl can be in equilibrium with the corresponding quinone system (I vs. II), which itself is reactive to thiol containing entities (Fig. 5B). Compound CM-568 lacks the internal hydrogen bonding system and the possibility to exist in the other quinonic form. As such, the types of thiols (cysteine groups) with which it can react will be different than CM-728. Regardless, the RSH-based mechanism in yeast is further supported by the selective loss of fluorescence for the GFP variants (CFP and YFP) relative to the DsRed variants (mCherry) (Fig. 4B and S6B); it is known that GFPs, but not mCherry, rely on the avoidance of disulfide bonded oligomerization for fluorescence [30].

As for CM-568, the most remarkable and differential phenotype was the appearance of refractive inclusions. Visible inclusions made of protein aggregates, lipid droplets or pyrophosphate deposits have been frequently reported in yeast under several stress conditions [31]. However, in most cases, these inclusions are small and spherical under BF microscopy, whereas in our case they were mostly thick and rod-shaped, frequently arranged as parallel bars (Fig. 4A). Such inclusions are rare, and are reminiscent of microtubule aggregates observed in yeast cells incubated under conditions that combine starvation with osmotic shock and phosphorus depletion [32]. Thus, CM-568 might lead to these very stressful physiological conditions. Alternatively, the oxazepine-NQ combination in this compound may cause microtubules to aggregate in that manner. This hypothesis is consistent with previous reports of tubulin as a major target of the oxazepine moiety [10]. Interestingly, CM-568 is expected to react with thiols like its close analogue CM-806 (Fig. 5B); however, the latter was inactive in yeast (Fig. 2). This indicates that there must be other chemical properties that render CM-568 active (e.g., target specificity, cell uptake ability, etc.).

Distinct physicochemical properties of CM-568 and CM-728 could also partly explain the different MOAs. Both compounds share similar lipophilicity (log *P*) and moderate water solubility (log *S*), and neither violates any of the five Lipinski's rules of druglikeness (Table S5). However, CM-728 is more polar and contains hydroxyl groups that can be ionized to alkoxy anions at alkaline pH (Fig. S8). This predicts that some of CM-728 could be targeted to peroxisomes through ion trapping. Peroxisomes play a central role in lipid metabolism and are also an important source of non-mitochondrial ROS [33]. To counteract ROS, peroxisomes house numerous antioxidant enzymes, including peroxiredoxins. Thus, it is tempting to speculate that yeast and humans may share the same CM-728 MOA. In support of this, we found that CM-728 generates ROS but is equally toxic with or without a functional mitochondrial respiratory chain (Fig. 2). Finally, it is worth mentioning that both compounds contain an aromatic amine (pyridyl) group that is protonated at the acidic pH found in yeast vacuoles. This suggests that yeast vacuoles may play a role in their differential toxicity relative to other non-toxic analogues without the pyridyl appendage. Perhaps, this appendage aids in their intracellular accumulation, or perhaps activating biotransformation events can occur in this organelle.

CM-568 and CM-728 showed similar cytotoxic potency in wild-type yeast (Fig. 1, Fig. 2), although CM-568 may be intrinsically more potent when the pleitropic drug resistance variable is removed from the equation (Fig. 2B and C). CM-728 is also cytotoxic against triple negative breast cancer cell (TNBC) lines, and has the potential to reduce orthopic TNBC tumours in mice [26]. Furthermore, CM-728 is cytotoxic against chronic myelogenous leukemia cells (McNaughton-Smith, manuscript in preparation). In the latter study, CM-728 was compared with CM-568, and CM-728 was 10-fold more potent. Thus, while the antitumor MOA of CM-728 could be further explored in yeast, CM-568 may be rather developed as a potential antifungal compound based on its unique MOA in *S. cerevisiae*. Future work will determine whether such a goal is worth pursuing.

## 5 Conclusion

Two fused oxazepine-naphthoquinones have been identified as cytotoxic to yeast by halo-based screening. These two compounds differ from other oxazepine-naphthoquinones in the screening in the presence of an electron withdrawing pyridyl appendage to the oxazepine ring. They in turn differ from each other in the presence of hydroxyl groups in the naphthoquinone moiety, which is sufficient to change their MOA from a ROS and/or redox cycling mechanism to one whose hallmarks are characteristic rod-shaped refractive inclusions. SAR studies suggest that both compounds react with thiol groups in cysteines through a different chemistry, encouraging further research on oxazepine-naphthoquinones as potential new drugs with diverse MOAs.

## CRediT authorship contribution statement

**Laura Anaissi-Afonso:** Visualization, Methodology, Investigation. **Silvia Santana-Sosa:** Visualization, Methodology, Investigation. **Isabel Lorenzo-Castrillejo:** Investigation. **Grant McNaughton-Smith:** Writing – review & editing, Visualization, Resources, Formal analysis. **Félix Machín:** Writing – review & editing, Writing – original draft, Visualization, Validation, Supervision, Resources, Project administration, Methodology, Investigation, Funding acquisition, Formal analysis, Conceptualization.

## Data availability statement

All experimental data is included in the article and supplementary material. CEAMED compounds are subjected to patent rights. Yeast strains are available upon request.

## Declaration of competing interest

The authors declare the following financial interests/personal relationships which may be considered as potential competing interests: Grant McNaughton-Smith reports a relationship with Centro Atlántico del Medicamento S.A. (CEAMED S.A) that includes: employment and equity or stocks. Grant McNaughton-Smith has patent #EP4177246 pending to Centro Atlántico del Medicamento S.A. (CEAMED S.A). The other authors declare that they have no known competing financial interests or personal relationships that could have appeared to influence the work reported in this paper.

## References

[1] Kumagai Y., Shinkai Y., Miura T., Cho A.K. (2012). The chemical biology of naphthoquinones and its environmental implications. Annu. Rev. Pharmacol. Toxicol..

[2] Fowler P., Meurer K., Honarvar N., Kirkland D. (2018). A review of the genotoxic potential of 1,4-naphthoquinone. Mutat. Res. Genet. Toxicol. Environ. Mutagen.

[3] Klotz L.O., Hou X., Jacob C. (2014). 1,4-naphthoquinones: from oxidative damage to cellular and inter-cellular signaling. Molecules.

[4] Shao Z., Wang H., Ren H., Sun Y., Chen X. (2023). The anticancer effect of napabucasin (BBI608), a natural naphthoquinone. Molecules.

[5] Qiu H.-Y., Wang P.-F., Lin H.-Y., Tang C.-Y., Zhu H.-L., Yang Y.-H. (2017). Naphthoquinones: a continuing source for discovery of therapeutic antineoplastic agents. Chem. Biol. Drug Des..

[6] Anaissi-Afonso L., Oramas-Royo S., Ayra-Plasencia J., Martín-Rodríguez P., García-Luis J., Lorenzo-Castrillejo I., Fernández-Pérez L., Estévez-Braun A., Machín F. (2018). Lawsone, juglone, and β-lapachone derivatives with enhanced mitochondrial-based toxicity. ACS Chem. Biol..

[7] Kapoor N., Kandwal P., Sharma G., Gambhir L. (2021). Redox ticklers and beyond: naphthoquinone repository in the spotlight against inflammation and associated maladies. Pharmacol. Res..

[8] Rani R., Sethi K., Gupta S., Varma R.S., Kumar R. (2022). Mechanism of action and implication of naphthoquinone as potent anti-trypanosomal drugs. Curr. Top. Med. Chem..

[9] Stefaniak M., Olszewska B. (2021). 1,5-Benzoxazepines as a unique and potent scaffold for activity drugs: a review. Arch. Pharm. (Weinheim).

[10] Mulligan J.M., Greene L.M., Cloonan S., Mc Gee M.M., Onnis V., Campiani G., Fattorusso C., Lawler M., Williams D.C., Zisterer D.M. (2006). Identification of tubulin as the molecular target of proapoptotic pyrrolo-1,5-benzoxazepines. Mol. Pharmacol..

[11] Brindisi M., Ulivieri C., Alfano G., Gemma S., de Asís Balaguer F., Khan T., Grillo A., Chemi G., Menchon G., Prota A.E., Olieric N., Lucena-Agell D., Barasoain I., Diaz J.F., Nebbioso A., Conte M., Lopresti L., Magnano S., Amet R., Kinsella P., Zisterer D.M., Ibrahim O., O'Sullivan J., Morbidelli L., Spaccapelo R., Baldari C., Butini S., Novellino E., Campiani G., Altucci L., Steinmetz M.O., Brogi S. (2019). Structure-activity relationships, biological evaluation and structural studies of novel pyrrolonaphthoxazepines as antitumor agents. Eur. J. Med. Chem..

[12] Schenone M., Dančík V., Wagner B.K., Clemons P.A. (2013). Target identification and mechanism of action in chemical biology and drug discovery. Nat. Chem. Biol..

[13] Simon J.A. (2001). Yeast as a model system for anticancer drug discovery. Expert Opin. Ther. Targets.

[14] Ho C.H., Piotrowski J., Dixon S.J., Baryshnikova A., Costanzo M., Boone C. (2011). Combining functional genomics and chemical biology to identify targets of bioactive compounds. Curr. Opin. Chem. Biol..

[15] Onge R. St, Schlecht U., Scharfe C., Evangelista M. (2012). Forward chemical genetics in yeast for discovery of chemical probes targeting metabolism. Molecules.

[16] Rodriguez C.E., Shinyashiki M., Froines J., Yu R.C., Fukuto J.M., Cho A.K. (2004). An examination of quinone toxicity using the yeast Saccharomyces cerevisiae model system. Toxicology.

[17] Rodrigues-Pousada C., Devaux F., Caetano S.M., Pimentel C., da Silva S., Cordeiro A.C., Amaral C. (2019). Yeast AP-1 like transcription factors (Yap) and stress response: a current overview. Microb. Cell.

[18] Rudner A.D., Murray A.W. (1996). The spindle assembly checkpoint. Curr. Opin. Cell Biol..

[19] Pizzul P., Casari E., Gnugnoli M., Rinaldi C., Corallo F., Longhese M.P. (2022). The DNA damage checkpoint: a tale from budding yeast. Front. Genet..

[20] Miyamoto Y., Machida K., Mizunuma M., Emoto Y., Sato N., Miyahara K., Hirata D., Usui T., Takahashi H., Osada H., Miyakawa T. (2002). Identification of Saccharomyces cerevisiae isoleucyl-tRNA synthetase as a target of the G1-specific inhibitor Reveromycin A. J. Biol. Chem..

[21] Matos-Perdomo E., Santana-Sosa S., Ayra-Plasencia J., Medina-Suárez S., Machín F. (2022). The vacuole shapes the nucleus and the ribosomal DNA loop during mitotic delays. Life Sci. Alliance.

[22] Quevedo O., García-Luis J., Lorenzo-Castrillejo I., Machín F. (2011). No role of homologous recombination in dealing with β-lapachone cytotoxicity in yeast. Chem. Res. Toxicol..

[23] Ramos-Pérez C., Dominska M., Anaissi-Afonso L., Cazorla-Rivero S., Quevedo O., Lorenzo-Castrillejo I., Petes T.D., Machín F. (2019). Cytological and genetic consequences for the progeny of a mitotic catastrophe provoked by Topoisomerase II deficiency. Aging (Albany. NY).

[24] Tsang C.K., Bertram P.G., Ai W., Drenan R., Zheng X.F.S.S. (2003). Chromatin-mediated regulation of nucleolar structure and RNA Pol I localization by TOR. EMBO J..

[25] Strich R. (2015). Programmed cell death initiation and execution in budding yeast. Genetics.

[26] Spínola-lasso E., Montero J.C., Jiménez-monzón R., Estévez F., Quintana J., Guerra B., Elokely K.M., León F., Rosario H., Fernández-pérez L. (2023). Chemical-proteomics identify peroxiredoxin-1 as an actionable target in triple-negative. Breast Cancer.

[27] Rhee S.G. (2016). Overview on peroxiredoxin. Mol. Cells..

[28] Bajor M., Zych A.O., Graczyk-Jarzynka A., Muchowicz A., Firczuk M., Trzeciak L., Gaj P., Domagala A., Siernicka M., Zagozdzon A., Siedlecki P., Kniotek M., O'Leary P.C., Golab J., Zagozdzon R. (2018). Targeting peroxiredoxin 1 impairs growth of breast cancer cells and potently sensitises these cells to prooxidant agents. Br. J. Cancer.

[29] Angulo-Elizari E., Henriquez-Figuereo A., Morán-Serradilla C., Plano D., Sanmartín C. (2024). Unlocking the potential of 1,4-naphthoquinones: a comprehensive review of their anticancer properties. Eur. J. Med. Chem..

[30] Suzuki T., Arai S., Takeuchi M., Sakurai C., Ebana H., Higashi T., Hashimoto H., Hatsuzawa K., Wada I. (2012). Development of cysteine-free fluorescent proteins for the oxidative environment. PLoS One.

[31] Barnett J.A., Robinow C.F. (2002). A history of research on yeasts 4: cytology part I, 1890-1950. Yeast.

[32] Tanaka K., Mizunaga T. (1974). Striated and crystalline inclusions in the nuclei and cytoplasm of intact yeast cells and yeast protoplasts. J. Ultrasructure Res..

[33] Fransen M., Nordgren M., Wang B., Apanasets O. (2012). Role of peroxisomes in ROS/RNS-metabolism: implications for human disease. Biochim. Biophys. Acta, Mol. Basis Dis..

