# Supplementary material for: Fused oxazepine-naphthoquinones as novel cytotoxic agents with diverse modes of action in yeast
Source: Heliyon. 2024 Dec 10;10(24):e41105. doi: 10.1016/j.heliyon.2024.e41105 (PMC11699245; doi:10.1016/j.heliyon.2024.e41105)
Supplement: Multimedia component 1 [file mmc1.pdf]

Fused oxazepine-naphthoquinones as novel cytotoxic agents with diverse modes of action in yeast

Laura Anaissi-Afonso[a,b], Silvia Santana-Sosa[a,b], Idaira Hueso-Falcón[c], Isabel Lorenzo-Castrillejo[a], Grant McNaughton-Smith[c], Félix Machín[a,b,d,*]

[a] *Unidad de Investigación, Hospital Universitario Nuestra Señora de Candelaria, Instituto de Investigación Sanitaria de Canarias (IISC), 38010 Santa Cruz de Tenerife, Spain.*

[b] *Instituto de Tecnologías Biomédicas, Universidad de La Laguna, 38200 San Cristóbal de La Laguna, Spain.*

[c] *Centro Atlántico del Medicamento S.A. (CEAMED S.A), 38204 La Laguna, Spain.*

[d] *Facultad de Ciencias de la Salud, Universidad Fernando Pessoa Canarias, 35450 Las Palmas de Gran Canaria, Spain.*

* Corresponding author. *Unidad de Investigación, Hospital Universitario Nuestra Señora de Candelaria, Ctra del Rosario 145, 38010 Santa Cruz de Tenerife, Spain.*

E-mail address: fmachin@fciisc.es (F. Machín).

Supplemental Figures and Tables

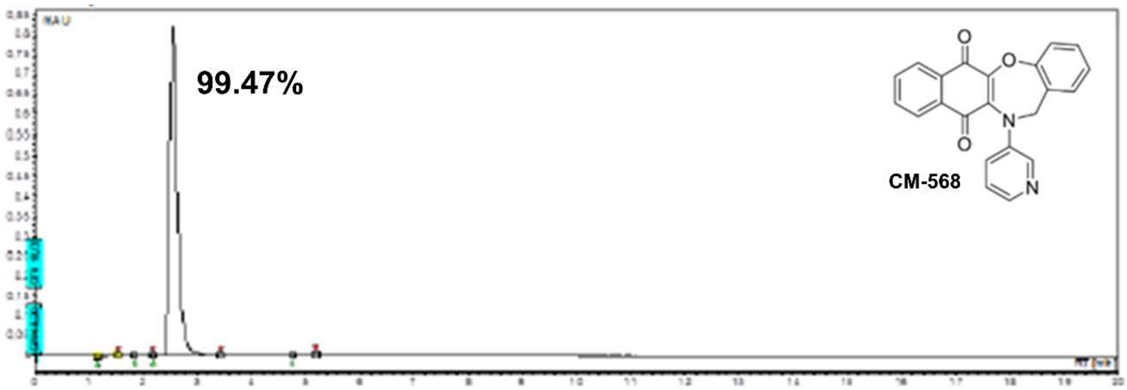

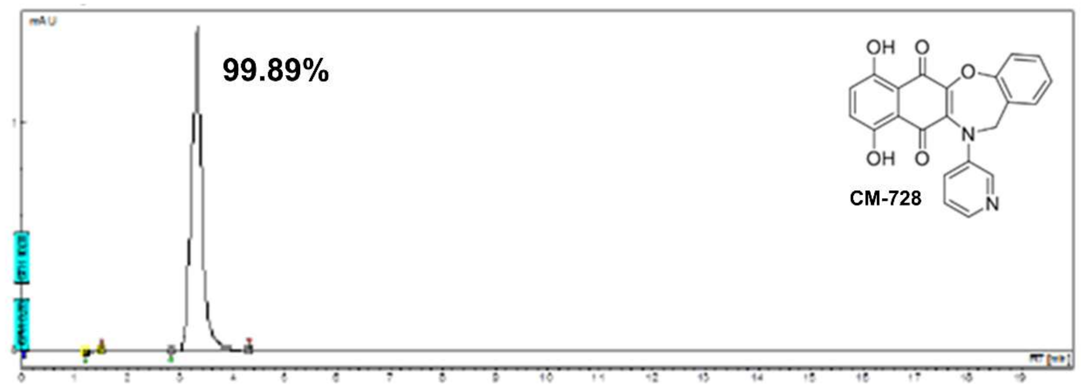

Figure S1. Purity assessment of CM-568 and CM-728. HPLC profiles of the corresponding compounds. Purity (percentage of the compound peak area) is included.

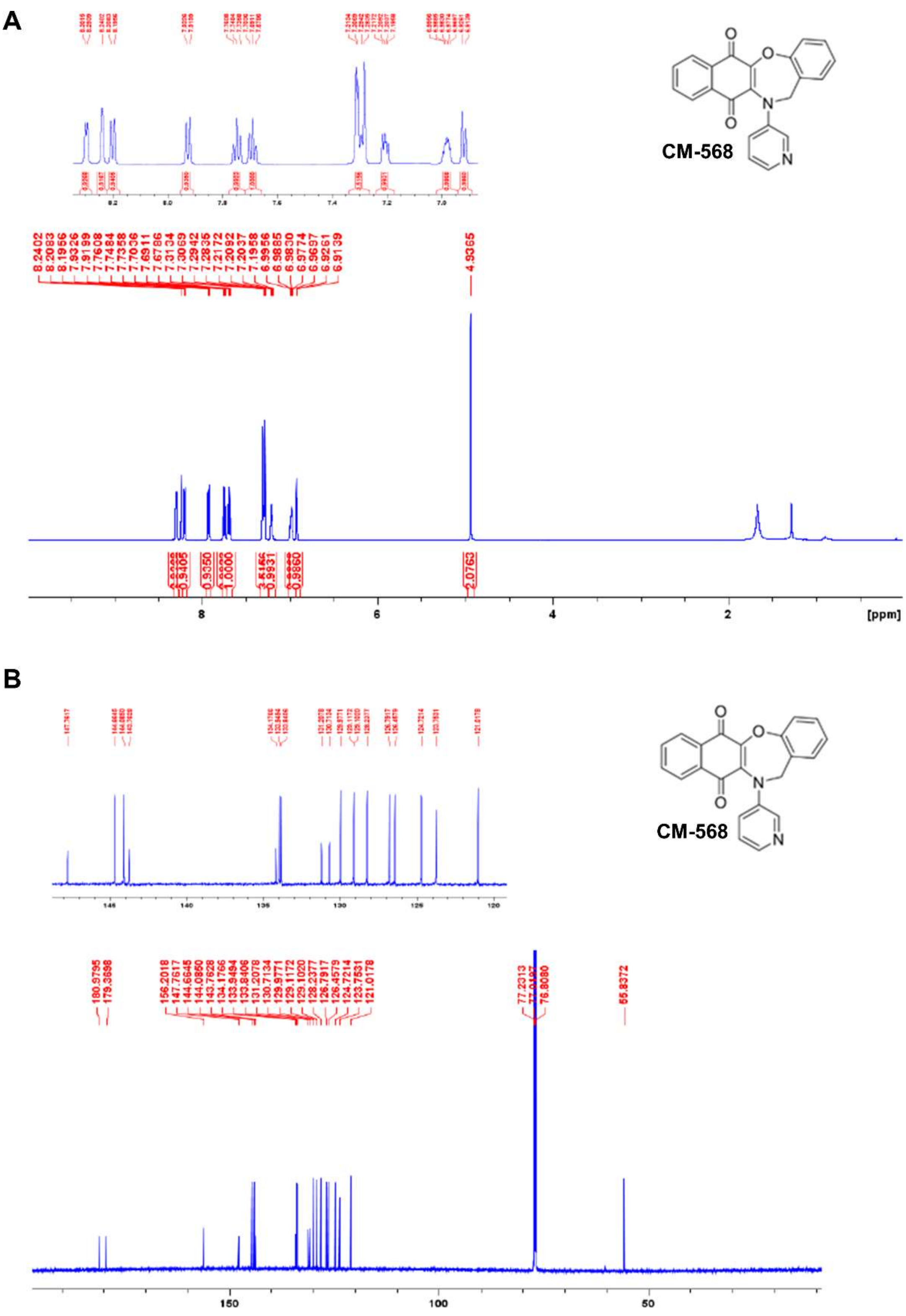

Figure S2. Structure determination of CM-568. CM-568 is 12-(Pyridin-3-yl)-12,13-dihydrobenzo[f]naphtho[2,3-b][1,4]oxazepine-6,11-dione. (**A**) ^{1}H NMR spectra (600 MHz, $CDCl_3$) δ: 8.27 (1H, d, J = 4.6 Hz), 8.21 (1H, s), 8.18 (1H, d, J = 7.6 Hz), 7.90 (1H, d, J = 7.6 Hz), 7.73 (1H, m), 7.67 (1H, m), 7.29-7.27 (3H, m), 7.18 (1H, m), 6.96 (1H, m), 6.90 (1H, J = 7.3 Hz), 4.91 (2H, s). (**B**) ^{13}C NMR spectra (150 MHz, $CDCl_3$) δ: 181.1 (s), 179.5 (s), 156.3 (s), 147.9 (s), 144.8 (d), 144.2 (d), 143.9 (s), 134.3 (s), 134.1 (d), 134.0 (d), 131.3 (s), 130.8 (s), 130.1 (d), 129.3 (s), 129.2 (d), 128.4 (d), 126.9 (d), 126.6 (d), 124.8 (d), 123.9 (d), 121.1 (d), 56.0 (t); EIMS: m/z 356 (24), 354 $[M]^+$ (100), 325 (47), 297 (53), 281 (50), 269 (47).

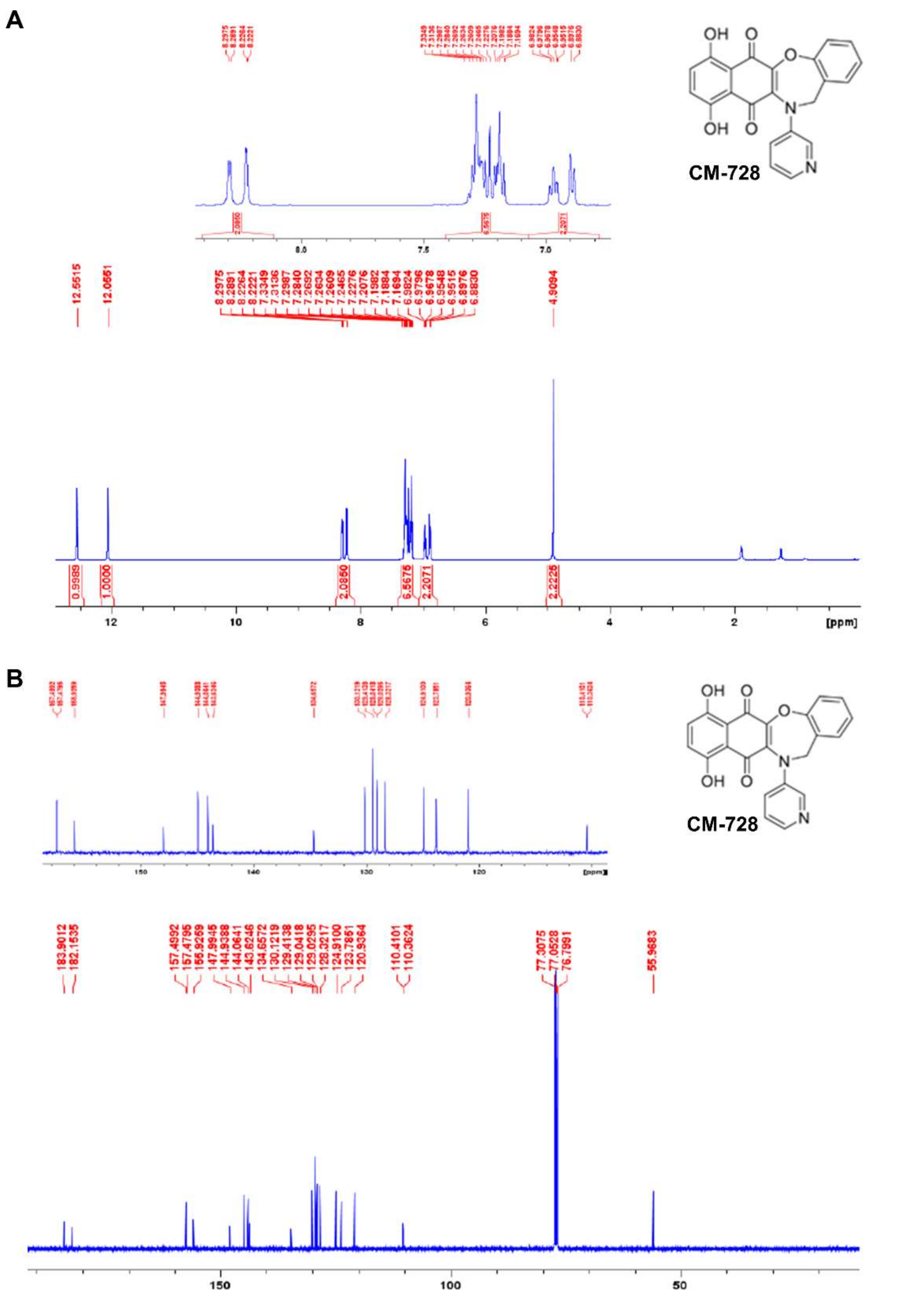

Figure S3. Structure determination of CM-728. CM-568 is 7,10-Dihydroxy-12-(pyridin-3-yl)-12,13-dihydrobenzo[f]naphtho[2,3-b][1,4]oxazepine-6,11-dione. (**A**) ^{1}H NMR spectra (500 MHz, $CDCl_3$) δ: 12.55 (1H, s, OH), 12.05 (1H, s, OH), 8.29 (1H, d, *J* = 4.2 Hz), 8.22 (1H, d, *J* = 2.1 Hz), 7.33-7.17 (6H, m), 6.96 (1H, ddd, *J* = 1.4, 7.3, 8.3 Hz), 6.89 (1H, d, *J* = 7.3 Hz), 4.91 (2H, s). (**B**) ^{13}C NMR spectra (125 MHz, $CDCl_3$) δ: 183.9 (s), 182.1 (s), 157.5 (s), 157.4 (s), 155. 9 (s), 148.0 (s), 144.9 (d), 144.1 (d), 143.6 (s), 134.6 (s), 130.1 (d), 129.4 (d, 2 x C), 129.0 (d), 129.0 (s), 128.3 (d), 124.9 (d), 123.8 (d), 120.9 (d), 110.4 (s), 110.3 (s), 55.9 (t).

A

Monoisotopic Mass, Odd and Even Electron Ions
61525 formula(e) evaluated with 13 results within limits (all results (up to 1000) for each mass)
Elements Used:
C: 22-22 H: 14-14 N: 0-2 O: 0-3
Tere TAG-
23Abr18-B 209 (11.860)

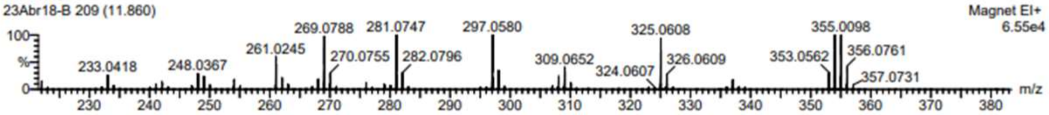

Minimum:	0.10				-1.5	
Maximum:	100.00		5.0	10.0	50.0	

Mass	RA	Calc. Mass	mDa	PPM	DBE	Formula
354.1035	43.23	354.1004	3.1	8.8	17.0	C22 H14 N2 O3
354.1002	55.94	354.1004	-0.2	-0.6	17.0	C22 H14 N2 O3
354.0970	75.22	354.1004	-3.4	-9.6	17.0	C22 H14 N2 O3
340.0996	0.29	340.0974	2.2	6.5	16.5	C22 H14 N O3
340.0965	0.43	340.0974	-0.9	-2.6	16.5	C22 H14 N O3
338.1035	0.10	338.1055	-2.0	-5.9	17.0	C22 H14 N2 O2
326.0970	3.93	326.0943	2.7	8.3	16.0	C22 H14 O3
326.0940	5.66	326.0943	-0.3	-0.9	16.0	C22 H14 O3
324.0996	0.19	324.1025	-2.9	-8.9	16.5	C22 H14 N O2
310.1018	1.27	310.0994	2.4	7.7	16.0	C22 H14 O2
310.0989	1.80	310.0994	-0.5	-1.6	16.0	C22 H14 O2
308.1088	0.21	308.1075	1.3	4.2	16.5	C22 H14 N O
308.1060	0.51	308.1075	-1.5	-4.9	16.5	C22 H14 N O

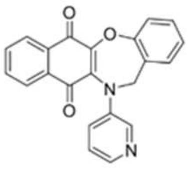

B

Monoisotopic Mass, Odd and Even Electron Ions
4145 formula(e) evaluated with 92 results within limits (all results (up to 1000) for each mass)
Elements Used:
C: 5-22 H: 5-14 N: 0-2 O: 0-5
Elisa
18-May16-CAFAMMA 96 (2.427)

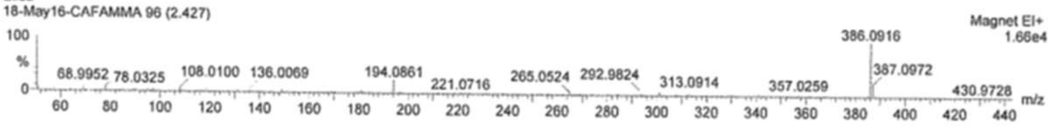

Minimum:	0.10				-1.5		
Maximum:	100.00		5.0	10.0	50.0		

Mass	RA	Calc. Mass	mDa	PPM	DBE	i-FIT	Formula
386.0916	100.00	386.0903	1.3	3.4	17.0	32.0	C22 H14 N2 O5
352.0583	0.19	352.0610	-2.7	-7.7	18.5	5546033.5	C22 H10 N O4
351.0320	0.58	351.0293	2.7	7.7	19.5	2773018.0	C22 H7 O5
341.0834	4.18	341.0814	2.0	5.9	16.5	2773199.5	C22 H13 O4
339.0793	1.28	339.0770	2.3	6.8	17.5	589.3	C21 H11 N2 O3
330.1016	0.62	330.1004	1.2	3.6	15.0	2773015.5	C20 H14 N2 O3
329.0928	3.01	329.0926	0.2	0.6	15.5	2.3	C20 H13 N2 O3
325.1005	0.51	325.0977	2.8	8.6	16.5	5546057.5	C21 H13 N2 O2
314.0969	2.60	314.0943	2.6	8.3	15.0	2773072.3	C21 H14 O3
311.0843	0.70	311.0821	2.2	7.1	16.5	985.4	C20 H11 N2 O2
309.0501	0.14	309.0511	-1.0	-3.2	13.5	2773075.8	C16 H9 N2 O5
308.0494	1.11	308.0473	2.1	6.8	18.0	2773026.5	C21 H8 O3
303.0918	0.23	303.0922	-0.4	-1.3	18.5	5546036.5	C22 H11 N2
		303.0895	2.3	7.6	14.0	5546035.0	C19 H13 N O3
301.0735	5.66	301.0739	-0.4	-1.3	15.0	5.3	C19 H11 N O3
297.0554	0.81	297.0552	0.2	0.7	16.5	5546079.5	C20 H9 O3
285.0318	2.03	285.0300	1.8	6.3	16.5	55.0	C17 H5 N2 O3
		285.0340	-2.2	-7.7	20.5	43.6	C22 H5 O
283.0260	0.54	283.0269	-0.9	-3.2	17.0	222.6	C18 H5 N O3
274.0648	0.52	274.0657	-0.9	-3.3	18.5	5546058.0	C21 H8 N
		274.0630	1.8	6.6	14.0	5546055.5	C18 H10 O3
271.0893	0.39	271.0871	2.2	8.1	14.5	104.1	C18 H11 N2 O
266.0601	0.88	266.0606	-0.5	-1.9	16.5	2773017.5	C19 H6 N O
		266.0579	2.2	8.3	12.0	2773016.5	C16 H10 O4
265.0524	4.32	265.0528	-0.4	-1.5	17.0	2.1	C19 H7 N O

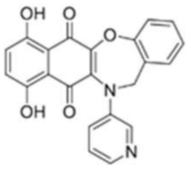

Figure S4. High resolution mass spectrometry (HRMS) of CM-568 and CM-728. Settings for multiple mass analysis: Tolerance = 10 ppm; DEB: min = -1.5, max = 50. (**A**) CM-568 HRMS: calcd. for $C_{22}H_{14}N_2O_3$ 354.1002, found 354.1008; mp: 190-192 °C. (**B**) CM-728 HRMS: calcd. for $C_{22}H_{14}N_2O_5$ 386.0903, found 386.0916; Anal. Calcd. for $C_{22}H_{14}N_2O_5$: C, 68.39; H, 3.65; N, 7.25; O, 20.71. Found: C, 68.45; H, 3.74; N, 7.43 %; mp: 214-215 °C.

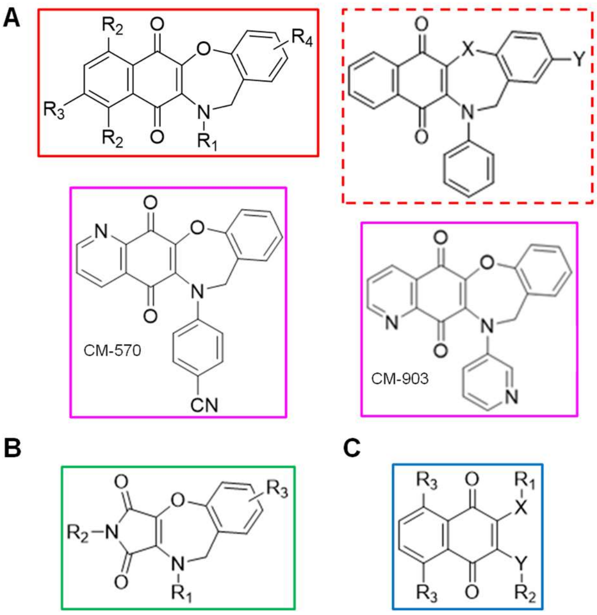

D

Plate #1	1	2	3	4	5	6
A	CM319	CM424	CM447	CM513	CM549	CM571
B	CM320	CM430	CM448	CM524	CM562	CM573
C	CM361	CM432	CM501	CM526	CM564	CM575
D	CM363	CM433	CM504	CM535	CM565	CM579
E	CM370	CM435	CM508	CM536	CM566	CM580
F	CM407	CM437	CM509	CM538	CM568	CM582
G	CM410	CM438	CM510	CM539	CM569	CM584
H	CM422	CM444	CM511	CM548	CM570	DMSO

Plate #2	1	2	3	4	5	6
A	CM587	CM599	CM648	CM670	CM700	CM725
B	CM588	CM600	CM649	CM674	CM706	CM728
C	CM590	CM601	CM650	CM676	CM712	CM732
D	CM591	CM602	CM654	CM678	CM715	CM738
E	CM593	CM621	CM658	CM683	CM718	CM733
F	CM594	CM625	CM660	CM685	CM720	CM745
G	CM596	CM643	CM661	CM686	CM721	CM755
H	CM598	CM645	CM669	CM689	CM723	DMSO

Plate #3	1	2	3	4	5	6
A	CM756	CM768	CM805	CM817	CM869	CM903
B	CM757	CM784	CM807	CM819	CM870	CM907
C	CM758	CM785	CM808	CM820	CM872	CM908
D	CM759	CM788	CM810	CM825	CM873	CM910
E	CM761	CM793	CM811	CM828	CM877	CM912
F	CM762	CM798	CM813	CM833	CM882	CM919
G	CM764	CM803	CM814	CM840	CM884	CM924
H	CM765	CM804	CM816	CM868	CM886	DMSO

Figure S5. Schematic of the compounds analysed and their position on the plates. (**A-C**) Schematic of the main compound families tested. (**A**) In solid red, oxazepine-naphthoquinones (oxa-NQ); in dashed red, diazepine-NQ; in solid pink, oxa-NQ derivatives where the NQ system has been converted to a quinoline-5,8-dione. (**B**) In green, oxazepine-pyrolediones. (**C**) In blue, oxa-NQ-like molecules where the oxazepine ring is broken. (**D**) Compounds included in this study and their position on three consecutive 96-well plates. Compound numbers are sorted first from top to bottom and then from left to right.

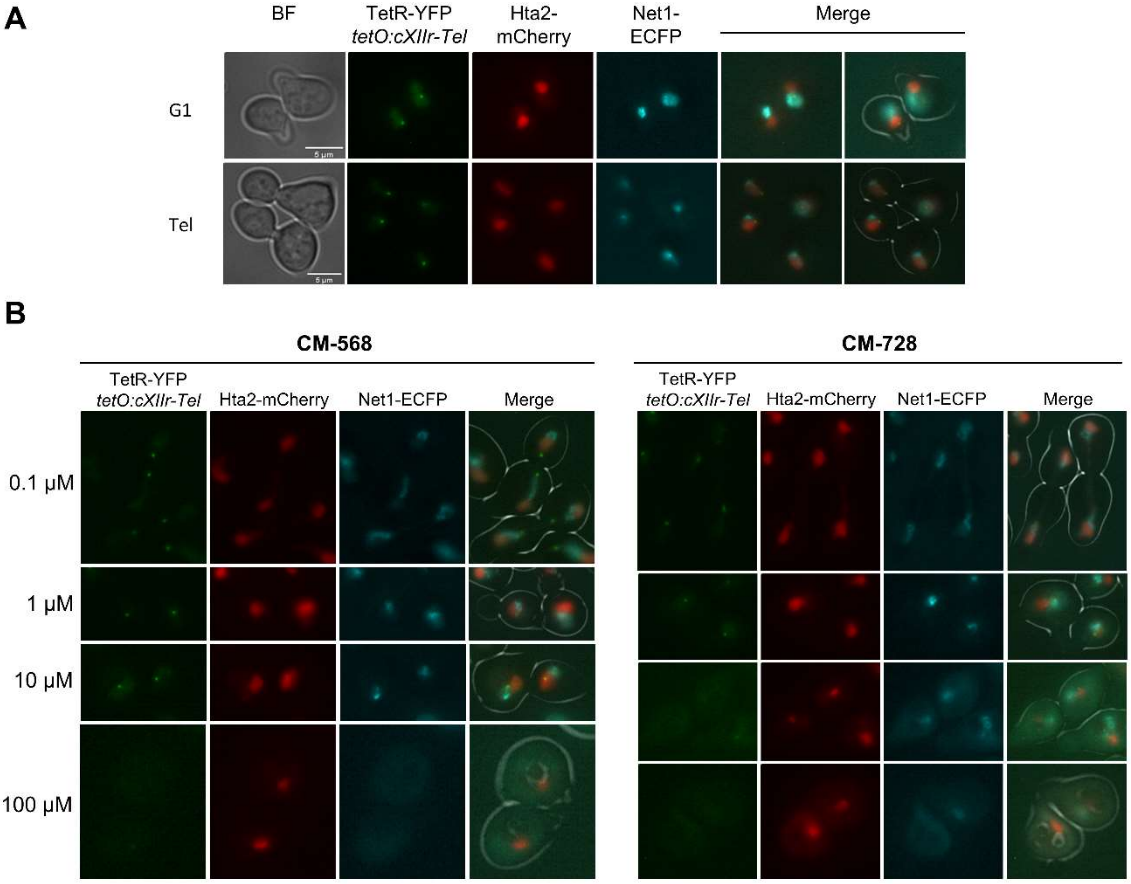

Figure S6. Effect of CM-568 and CM-728 in the strain engineered to report cell cycle, nucleolar and nuclear compaction. (**A**) Representative G1 and telophase block micrographs. The strain FM2707 (*MATa Δbar1 cdc15-2 ADH1p-OsTIR1 TetR-YFP cXIIr-Tel(1061Kb):tetO(x224) HTA2-mCherry NET1-eCFP*) was arrested in G1 for 3h at 25 ºC and then released to reach and get blocked in telophase at 34 ºC in the presence of DMSO 1% v/v. In the BF channel, note the characteristic pear-shaped cell morphology of G1 arrest by α-factor pheromone. In telophase, the cell appears with a dumbbell morphology with the nuclear material fully segregated, including the nucleolus and the rDNA-bearing chromosome arm; the mother cell in the dumbbell retains the pear shape. (**B**) Fluorescence decay after 3h incubation with the corresponding drug concentrations. Note that at 10 µM there is a loss of CFP and YFP fluorescence for CM-728 but not for CM-568. At 100 µM there is no CFP/YFP fluorescence for either compound, but mCherry is not affected.

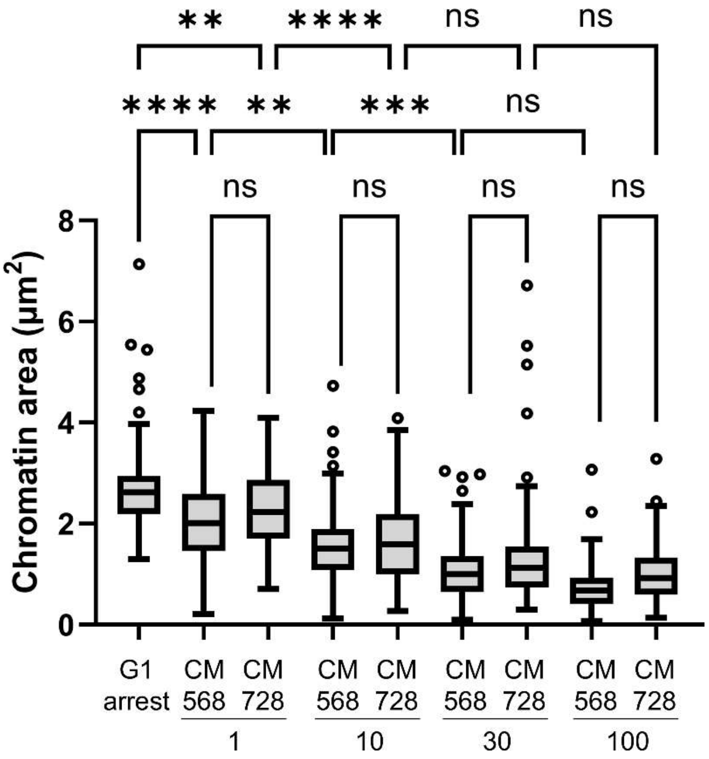

Figure S7. Chromatin compaction with increasing concentrations of CM-568 and CM-728. This chart is as in Figure 4E but with statistical comparisons included after a one-way ANOVA followed by a Tukey's post hoc test. Only meaningful comparisons are included; i.e., for each compound, one concentration with the next higher, and between compounds for the same concentration (n.s., $p>0.05$; **, $p<0.01$; ***, $p<0.001$; ****, $p<0.0001$).

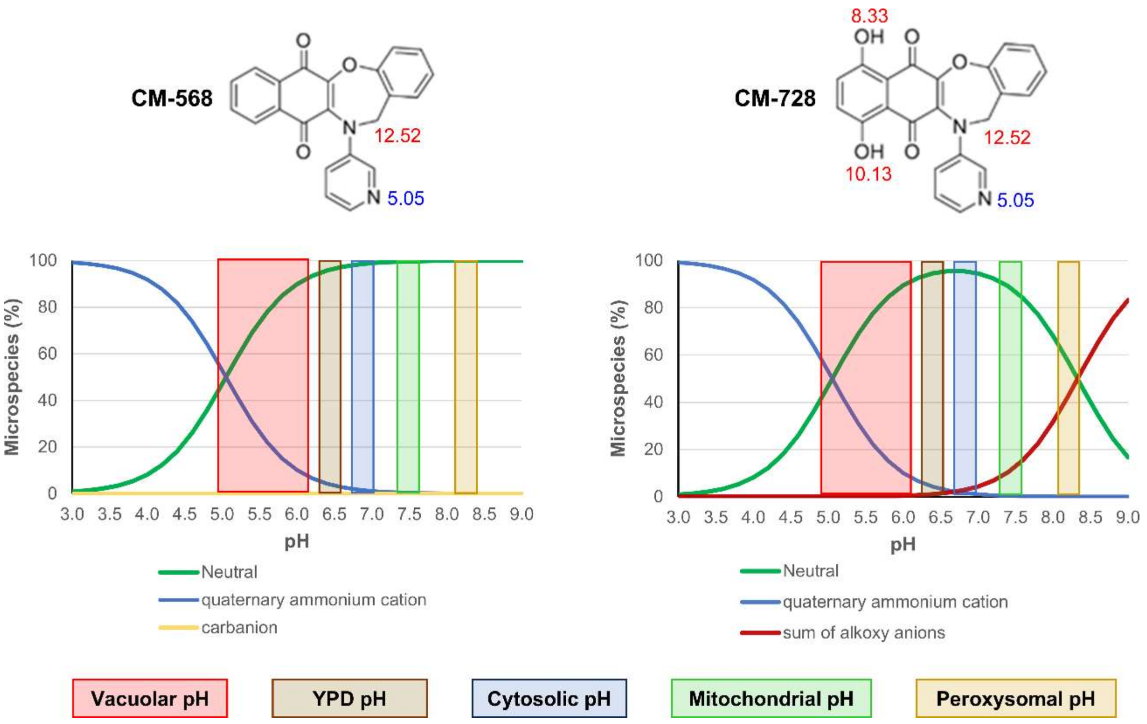

Figure S8. Distribution of ionized microspecies of CM-568 and CM-728 as function of pH. On the top, the pKa for each ionizing group in CM-568 and CM-728 is indicated; blue numbers, pKa of the group that can gain a H^+ ($-N=$ to $-NH^+=$), red numbers, pKa of groups that can lose a H^+ ($-OH$ to $-O^-$; $-CH_2-$ to $-CH^--$). Below, distribution of non-ionized and ionized species in the physiological pH range (3.0 – 9.0). In this range, only neutral (-N= and -OH), quaternary ammonium cation ($-NH^+=$), and alkoxy anion ($-O^-$) forms are significant. The boxes on the charts delimit the pH range in the fresh extracellular YPD media (pH ~ 6.2 - 6.5), the yeast cytosol (pH ~ 6.8 - 7.0), the yeast mitochondrial lumen (pH ~ 7.5), the yeast peroxisome (pH ~ 8.2), and the yeast vacuole (pH ~ 5.0 - 6.2). Note that, whereas both compounds are predicted to be targeted to the vacuole through ion trapping of the quaternary ammonium cation form, CM-728 could also get trapped within peroxisomes (and to a lesser extent in mitochondria) as alkoxy anions. The protonation/deprotonation analysis was performed by the Marvin Suite software (https://chemaxon.com/). The pH values for the boxes were obtained at https://bionumbers.hms.harvard.edu/search.aspx, and are a consensus of multiple publications.

Table S1. Oxazepine-naphthoquinones included in the yeast screen (see Figure S5A for the schematics, solid lines only).

CM	R1[a]	R2[b]	R3	R4	Activity[c]
**562**	-Ph	H	H	H	+
**564**	$-CH_2Ph$	H	H	H	-
**565**	Ph-4CN	H	H	H	++
**566**	-nBu	H	H	H	-
**568**	3-py	H	H	H	+++
**569**	2-py	H	H	H	+++
**570**	Ph-4CN	-	H	H	+
**571**	Ph-4CN	H	H	2-OMe	+
**573**	Ph-4CN	H	OH	H	-
**643**	Ph-4CONH$_2$	H	H	H	-
**660**	Ph-4SO2NH$_2$	H	H	H	-
**718**	Ph-4-CN	OH	H	H	+
**720**	Ph-4CONH$_2$	OH	H	H	+
**723**	Ph-4-CN	OH	H	4F	++
**725**	Ph-4-SO2NH$_2$	OH	H	H	-
**728**	3-Py	OH	H	H	+++
**732**	Ph-4CONH$_2$	OH	H	4F	-
**733**	Ph-4CONH2	OH	H	2OMe	+
**738**	Ph	OH	H	H	-
**755**	Ph-4CO$_2$Me	OH	H	H	+
**756**	Ph-4CO$_2$H	OH	H	H	-
**758**	Ph-4COmorp	OH	H	H	-
**759**	3-Py-4-morp	OH	H	H	-
**764**	3-Py-4-NCH$_3$(CH$_2$)$_2$OH	OH	H	H	-
**765**	3-Py-4-N(CH$_3$)$_2$	OH	H	H	-
**784**	3-Py	OH	H	CO$_2$Me	+
**785**	3-Py	OH	H	CO$_2$H	-
**793**	Ph	OMe	H	H	-
**798**	Ph-2-CN	OMe	H	H	-
**808**	3-Py-4-morp	OMe	H	H	-
**833**	3-Py-4-NMP	OH	H	H	-
**903**	3-Py	-	H	H	-

[a] Ph, phenyl; Py, pyridyl; morp, morpholino; NMP, N-methylpiperazine; nBu, n-butyl.

[b] CM-570 and CM-903 lack the naphthoquinone moiety, having a quinoline-5,8-dione instead (see structures in Figure S5A). As a result, no R2 is present it is denoted as - in the table).

[c] Activity is based on the size and depth of the inhibition halo in the wild type yeast strain BY4741. Growth was normalized so that DMSO equals 1 and an inhibition halo that cover the surrounding spots equals 0. Thus, -, normalized growth (NG) >0.8; +, $0.6 < NG < 0.8$; ++, $0.4 < NG < 0.6$; +++, $NG < 0.4$ (see Figure 1C for the plot of actual values).

Table S2. Diazepine-naphthoquinones derivatives in which the O atom in the oxazepine ring has been replaced by N groups (see Figure S5A for the schematic, dashed line).

CM	X	Y	Activity
**813**	NH	H	-
**817**	NMe	Cl	-

Table S3. Oxazepine-pyrroelediones included in the yeast screen (see Figure S5B for the schematic, solid green line).

CM	R1	R2	R3	Activity
**579**	Ph-4CN	$PhCH_2$	H	-
**587**	Ph-4CN	Ph	H	-
**588**	3-py	Ph	H	-
**590**	Ph-4CN	nBu	H	-
**591**	Ph-4CN	Ph-4CN	H	-
**593**	Ph	nBu	H	-
**594**	Ph	$CH_2CO_2CH_3$	H	-
**596**	nBu	$PhCH_2$	H	-
**600**	Ph-4CN	CH2CH2OH	H	-
**625**	3-py	$PhCH_2$	H	-
**654**	3-py	nBu	H	-
**658**	Ph-4SO_2NH_2	$PhCH_2$	H	-
**661**	Ph-4SO_2NH_2	nBu	H	-
**669**	Ph-4CN	Cyclopropyl	H	-
**674**	Ph-4$CONH_2$	H	H	-
**676**	Ph-4$CONH_2$	CH_3	H	-
**706**	3-py	$PhCH_2$	4Cl	-
**712**	Ph-4SO_2NH_2	nBu	4F	-

Table S4. Naphthoquinones derivatives with substitutes that resemble an open oxazepine-like ring (see Figure S5C for the schematic, solid blue line).

CM	X	Y	R1	R2	R3	Activity
**768**	NH	NH	Ph	Ph-4Cl	H	-
**788**	NH	NH	Ph	Ph	H	-
**803**	O	O	Ph	Ph	H	-
**807**	NH	O	Ph	Ph	H	-
**814**	NH	NH	Ph	Ph-4CN	H	-
**819**	NCH_3	NH	Ph	Ph	OH	-
**825**	NH	NH	Ph	Ph	OH	-
**828**	O	O	Ph	Ph	OH	-
**840**	O	NH	Ph	3-py	OH	-
**907**	O	NH	Ph	3-py-4-NMP	OH	-
**910**	CH_2	CH_2	Ph	Ph	H	-
**912**	-	-	Ph	Ph	H	-
**919**	CH_2	NH	Ph	3-py	H	-
**924**	-	NH	Ph	3-py	H	-

Table S5. Predicted physiochemical properties [a]:

CM	Log P (consensus)	tPSA	Log S (ESOL)	Rotable Bonds	Lipinski Violations
**728**	2.41	99.96	-5.13	1	0
**568**	2.85	59.50	-4.72	1	0

[a] Calculated using the SwissADME web service http://www.swissadme.ch/index.php

[b] Lipophilicity (Log $P_{o/w}$). Values are the consensus (average) of five predictions (iLogP, xLogP3, wLogP, mLogP, and SILICOS-IT).

[c] Topological Polar surface area (A^2).

[d] Water solubility (Log *S* by ESOL method). Class: Modelarely soluble ($-6 < \text{Log S} < -4$).

[e] Number of violations of the five Lipinski's rules (MW<500; mLogP ≤ 4.15; N or O ≤ 10; N or O ≤ 5)
